# Association of physical activity and sedentary time with blood cell counts: National Health and Nutrition Survey 2003-2006

**DOI:** 10.1371/journal.pone.0204277

**Published:** 2018-09-25

**Authors:** Erik A. Willis, Joseph J. Shearer, Charles E. Matthews, Jonathan N. Hofmann

**Affiliations:** 1 Metabolic Epidemiology Branch, Division of Cancer Epidemiology and Genetics, National Cancer Institute, Bethesda, Maryland, United States of America; 2 Cancer Prevention Fellowship Program, Division of Cancer Prevention, National Cancer Institute, Bethesda, Maryland, United States of America; 3 Occupational and Environmental Epidemiology Branch, Division of Cancer Epidemiology and Genetics, National Cancer Institute, Bethesda, Maryland, United States of America; Universidad Europea de Madrid, SPAIN

## Abstract

**Objective:**

To assess the association of objectively measured levels of physical activity and sedentary time with major blood cell counts (e.g. white blood cells, red blood cells, platelets) among adults.

**Methods:**

Data collected from the 2003–2004 and 2005–2006 cycles of the National Health and Nutrition Examination Survey (NHANES) was used to assess blood cell counts in relation to objectively measured physical activity and sedentary time (accelerometer). A series of linear regressions modes were used to assess these associations adjusting for a range of factors known to be associated with blood cell counts, including age, body mass index, dietary factors, and previous infections.

**Results:**

Higher levels of moderate-vigorous physical activity (p_trend_<0.001) and lower sedentary time (p_trend_ = 0.040) were associated with lower white blood cell counts.

**Conclusion:**

These results suggest that modifiable health behaviors, such as physical activity and sedentary time, may be associated with inflammatory status through white blood cell counts, which may be important for future disease risk.

## Introduction

Moderate-vigorous physical activity (MVPA) represents a modifiable health behavior that has been associated with reduced overall mortality and lower risk of many chronic disorders including cardiovascular disease, type-2 diabetes, and some cancers.[1] A prospective investigation of over 1.4 million individuals identified 13 specific cancer types that show a reduced risk with increased levels of MVPA [2]. While multiple mechanisms have been suggested to explain the beneficial effects of MVPA on cancer risk ranging from endocrine factors to immune function [3, 4], the precise mechanisms remain to be fully understood.

Emerging evidence has also suggested that increased sedentary time, independent of MVPA, is associated with increased risk for many of the same outcomes linked to low MVPA including increased overall mortality, type-2 diabetes, and cardiovascular disease [5]. While less research has been done investigating the mechanistic details of sedentary time and disease risk, increased inflammatory biomarkers such as C-reactive protein have been observed with more sedentary time [6], suggesting an important role for circulating inflammatory markers.

The complete blood count (CBC) is a clinical tool used to assess overall health status by measuring major blood components (e.g. white blood cells, red blood cells, platelets). Abnormal CBC results frequently accompany patients who are diagnosed with chronic illnesses [7]. Results from the CBC are not only helpful in diagnosis, but may provide insights into disease etiology. Increased pre-diagnostic levels of white blood cells (WBCs) [8–11] and platelets [12] have been shown to be associated with risk of developing certain malignancies (e.g. lung and gastric cancer) in prospective studies. Furthermore, high baseline WBCs levels have been associated with cancer mortality and overall mortality[13, 14]. While less is known about the effect of red blood cell (RBC) counts for cancer, an increased risk of other chronic disorders, such as coronary heart disease, has been observed among those with elevated RBC counts [15]. Due to the relationship of blood cell populations with chronic diseases, it would be interesting to know how physical inactivity influences blood cell populations.

The goal of the present analysis was to examine the association between MVPA and sedentary time with blood counts in a nationally representative sample of U.S. adults using the National Health and Nutrition Examination Survey (NHANES), with the hypothesis that increased MVPA and decreased sedentary time would be associated with healthier blood profiles.

## Materials and methods

NHANES is a cross-sectional study that uses a complex, multistage probability design to obtain a representative sample of the U.S. civilian noninstitutionalized population. The survey consists of a household interview and an examination conducted in a mobile examination center. Detailed descriptions of study design and methods are provided elsewhere [16, 17]. Subject data for this cross-sectional study were pooled from the 2003–2004 and 2005–2006 NHANES cycles because of the availability of the accelerometer measures.

### Study population

The overall study population was 20,470 individuals. We excluded participants <20 years of age and pregnant women, leaving 7,497 individuals (eligible sample). Those with missing CBC values, covariates, and insufficient accelerometer data were further excluded. Thus, data from 4,857 adults were available for analyses.

### Blood count analysis

Collection protocols and quality assurance for the CBC were conducted in accordance with published standard operating procedures [18]. Briefly, blood samples were collected from the arm of each individual participant and analyzed at the mobile examination centers by a certified medical technologist or phlebotomist.

### Physical activity measurements

Physical activity was assessed by a portable accelerometer (Actigraph 7164; Actigraph, LLC, Fort Walton Beach, FL) given to participants during their mobile examination center visit. The Actigraph accelerometer is a small, lightweight (5.1 × 4.1 × 1.5 cm; 0.4 kg), instrument that records vertical accelerations as an activity count, which provides an objective estimate of the intensity of bodily movement [19]. Accelerometers were worn on an elastic belt over the right hip during waking hours (except for water-based activities) for seven consecutive days. The data collection interval was set at one-minute epochs, and a minimum of 10 hours of wear time constituted a valid monitored day. Non-wear time was identified as ≥60 consecutive minutes with 0 counts per minute (cpm), with allowance for 1–2 minutes of accelerometer counts between 0 and 100 [20]. One valid day was required to be included in the analysis. Sedentary time was classified as worn time with accelerometer counts <100, and MVPA was classified using two established accelerometer count cut points (≥760 cpm, ≥2020 cpm) [20, 21]. We conducted analyses using both sets of cutoffs and obtained qualitatively similar results. For clarity of presentation, we present the accelerometer count ≥2020 results and provide results for the 760 threshold in supplemental materials (S1 and S2 Tables). To account for the influence of variation of wear time on both sedentary time and MVPA, wear time was included as a covariate in all analyses. Sedentary time and MVPA were examined as quartiles.

### Covariates

Interviewer-administered questionnaires were used to gather sociodemographic information. Smoking status was categorized according to serum-cotinine levels. Dietary intake was assessed by a 24-hour diet-recall coupled with U.S. Department of Agriculture food composition data. Data obtained from 24-hour recall were used to calculate diet quality index scores using the Healthy Eating Index-2015 (HEI-2015) [22]. The HEI-2015 is a measure of diet quality developed by the U.S. Department of Agriculture that assesses conformance to the Dietary Guidelines for Americans [23]. The HEI-2015 provides a point value based on how well a person meets the dietary guidelines, expressed as a percent per 1,000 kcals. Dichotomous variables were generated (yes/no) from self-reported medical history for asthma, anemia, arthritis, blood donation or transfusion, cancer, and illness past 30 days (flu, pneumonia, ear infection).

### Statistical analysis

Age (years) at the time of the survey was treated continuously. Categories of educational attainment, marital status, poverty–income ratio, sex, and race/ethnicity (non-Hispanic white, non-Hispanic black, Mexican American, and other) were used. Body mass index (BMI) was calculated as the weight of the person in kilograms divided by the square of height in meters and categorized into underweight (BMI <18.5 kg/m^2^), healthy weight (BMI = 18.5–24.9 kg/m^2^), overweight (25.0–29.9 kg/m^2^), and obese (BMI ≥ 30.0 kg/m^2^). To account for possible seasonal variation in physical activity and blood cell counts assessment date was dichotomized into six-month time periods when the examination was performed (November 1 through April 30, May 1 through October 31).

To obtain population-representative findings, we incorporated weights for sampling strata, primary sampling unit, and individual sampling weights for descriptive and inferential statistical analyses. Two-year sample weights for each NHANES cycle were combined to provide four-year weights for the 2003–2006 survey periods. Examination weights for our full sample were reweighted using an established method specific for individuals in NHANES with physical activity monitoring to account for the non-random absence of accelerometer data [24]. Linear regression analyses examined the associations of total sedentary time and MVPA with blood cell counts. Model 1 adjusted only for age, sex, race/ethnicity, and accelerometer wear time. Model 2 additionally adjusted for time of year of assessment, total sedentary time or MVPA as continuous covariates, as appropriate, as well as relevant confounding behavioral and medical factors identified through backward elimination (p < 0.2 for retention or induced >20% change in parameter estimates; Table 1). For cases where residual plots showed evidence of violation of the normality assumption box cox procedures were implemented and outcome variables were transformed. Results are reported as adjusted means for each quartile of exposure, back-transformed. Linear contrast tests were used to examine the linear trends across different physical activity and sedentary time quartiles.

**Table 1:** Covariates retained through backward elimination in fully adjusted models.

Blood Cell Types	Covariates retained
White blood cell count (1000 cells/μL)	HEI-2015 score, illness past 30 days (flu, pneumonia, or ear infection), asthma, donated blood, poverty income ratio, BMI, smoking status, marital status
Red blood cell count (million cells/μL)	HEI-2015 score, asthma, anemia, blood transfusion, arthritis, cancer or malignancy, poverty income ratio, BMI, marital status, smoking status
Platelet count (1000 cells/μL)	HEI-2015 score, illness past 30 days (flu, pneumonia, or ear infection), donated blood, asthma, blood transfusion, arthritis, cancer or malignancy, BMI, marital status, smoking status, poverty income ratio

HEI = healthy eating index. BMI = Body Mass Index (weight(kg)/height(m2). All models adjusted for sex, age, race/ethnicity (non-Hispanic white, Mexican American, non-Hispanic black, other) and total sedentary time and MVPA, as appropriate.

Interaction terms were added to the model to determine whether associations between physical activity and sedentary time quartiles varied by sex, race/ethnicity, age, or BMI (adjusting using Model 2, but without weighting, to avoid inflated standard errors). Stratified analysis differed from main analyses in that age was dichotomized into groups (<50 and ≥ 50 years). Race/ethnicity comparisons excluded the ‘Other’ race/ethnicity group and BMI comparison excluded “Underweight” group, where the small sample size precluded meaningful interpretation. All main effects, interactions, and trends were considered significant at a p<0.05. All analyses were conducted using PROC SURVEYFREQ, SURVEYMEANS, and SURVEYREG procedures implemented in SAS version 9.4 (SAS Institute Inc., Cary, NC).

## Results

Participant characteristics are presented in Table 2. The participant characteristics of the full sample were similar to all potentially eligible participants (Table 2). The average age of the full sample was 43.1 ± 0.8 years, 43% of participants were female. Sixty-seven percent of the sample were overweight or obese (BMI ≥ 25 kg/m^2^). On average, 5.3 ± 1.8 valid days of accelerometer data with approximately 14.7 hours/day of wear time were available. Mean time spent in MVPA was 25.7 ± 1.1 minutes/day with 515.6 ± 7.5 minutes/day in sedentary pursuits. Characteristics of accelerometer and blood counts are shown in Table 3.

**Table 2:** Population-weighted characteristics of the eligible sample and the full sample of U.S. adults ≥ 20 years (NHANES 2003–2006).

	Eligible Sample				Full Sample			
	N	Mean	95% CL		N	Mean	95% CL	
Age (years)	7497	43.2	42.5	43.9	4857	43.1	42.3	43.9
HEI Total Score	6873	54.9	54.3	55.5	4857	54.9	54.1	55.7
	**N**	**%**			**N**	**%**		
Female	2692	41.9			1827	43.2		
Race/Ethnicity								
*Non-Hispanic White*	3762	70.9			2520	72.2		
*Non-Hispanic Black*	1693	11.6			1020	10.5		
*Mexican American*	1479	8.3			973	8.3		
*Other*	563	9.2			344	9.0		
Education attainment								
*< Year 12*	2009	16.6			1208	15.2		
*Year 12 or equivalent*	1794	25.1			1148	24.4		
*Post high school*	3683	58.3			2501	60.4		
Marital Status								
*Married/living with partner*	4786	66.9			3234	68.8		
*Widowed*	330	2.3			191	2.2		
*Separated/divorced*	942	12.4			610	12.2		
*Never married/single*	1432	18.4			822	16.9		
Poverty Income Ratio								
*Quartile 1 (0 to 1*.*29)*	1857	17.9			1225	17.3		
*Quartile 2 (1*.*30 to 2*.*52)*	1811	21.7			1204	21.5		
*Quartile 3 (2*.*53 to 4*.*39)*	1738	28.2			1215	28.7		
*Quartile 4 (>4*.*40)*	1705	32.1			1213	32.5		
Smoking (serum cotinine)								
*non*, *< 10 ng/dL*	5176	69.8			3474	69.9		
*light*, *10–<100ng/dL*	513	6.8			363	7.3		
*moderate*, *100–<300 ng/dL*	988	14.8			647	14.6		
*heavy*, *≥300 ng/dL*	574	8.7			373	8.3		
BMI Category								
*Underweight (<18*.*5 kg/m*^*2*^*)*	230	3.0			69	1.6		
*Healthy weight (18*.*5 to 24*.*9 kg/m*^*2*^*)*	2115	31.2			1417	31.6		
*Overweight (25*.*0–29*.*9 kg/m*^*2*^*)*	2520	33.2			1728	33.4		
*Obese (≥30*.*0 kg/m*^*2*^*)*	2386	32.6			1643	33.3		
Currently have flu, pneumonia, ear infection	319	4.7			231	4.6		
Donated blood in past 12 months	295	5.7			236	6.1		
Doctor told asthma	900	13.6			585	13.2		
Treated for anemia/past 3 months	209	2.1			123	2.1		
Ever receive blood transfusion	786	9.7			520	9.2		
Doctor told arthritis	1530	19.8			1059	19.6		
Ever had cancer or malignancy	528	6.7			364	6.7		

CL = confidence limits. HEI = health eating index. BMI = body mass index (weight(kg)/height(m)^2^)

**Table 3:** Average accelerometer and blood cell counts of the full sample of U.S. adults ≥ 20 years (NHANES 2003–2006).

	Full Sample (n = 4857)		
	Mean	95% CL	
**Accelerometer variables**			
Wear time (minutes/day)	880.5	875.6	885.4
Average Intensity (cpm)	341.8	334.3	349.3
**MVPA**			
*Quartile 1 (≤ 7*.*0 minutes/day)*	3.6	3.4	3.7
*Quartile 2 (7*.*1 to 17*.*5 minutes/day)*	12.0	11.7	12.2
*Quartile 3 (17*.*6 to 34*.*3 minutes/day)*	25.3	24.9	25.7
*Quartile 4 (≥34*.*4 minutes/day)*	56.9	55.3	58.5
**Sedentary time**			
*Quartile 1 (≤ 389 minutes/day)*	318.5	314.8	322.3
*Quartile 2 (390 to 472 minutes/day)*	432.9	431.4	434.3
*Quartile 3 (473 to 553 minutes/day)*	511.1	509.3	512.9
*Quartile 4 (≥554 minutes/day)*	623.1	617.1	629.2
**Blood Cell Types**			
White blood cell count (1000 cells/μL)	7.3	7.2	7.4
Red blood cell count (million cells/μL)	4.8	4.8	4.8
Platelet count (1000 cells/μL)	273.4	270.9	276.0

MVPA = moderate-vigorous physical activity. MVPA using ≥2020 accelerometer count threshold. cpm = counts per minute.

### Associations of MVPA with blood cell counts

In model 1, after adjusting for age, sex, and race/ethnicity, there were significant linear associations of higher MVPA with lower counts of WBCs (p_trend_ <0.001) and platelets (p_trend_ = 0.003). In model 2, after additional adjustment for time of year of assessment, sedentary time, plus covariates, higher MVPA was only associated with lower counts of WBCs (p_trend_<0.001). Specifically, a 7% lower average count of WBCs was observed in individuals with high vs. low MVPA (Q4 vs. Q1 mean (95% CI): 7.1 (6.7–7.6) vs. 7.6 (7.1–8.2). Results are presented in Table 4. This significant inverse association of MVPA and WBCs was also observed using the 760 cpm cut point for MVPA (S2 Table)

**Table 4:** Adjusted means (95% CL) for continuous hematologic variables across quartiles of MVPA in U.S. adults ≥ 20 years (NHANES 2003–2006).

	MVPA												
	Quartile 1			Quartile 2			Quartile 3			Quartile 4			p_trend_
	Mean	95% CL		Mean	95% CL		Mean	95% CL		Mean	95% CL		
White blood cell count (1000 cells/μL)													
Model 1^a^	7.3	6.8	7.8	7.0	6.6	7.5	6.6	6.2	7.1	6.4	6.0	6.8	<0.001
Model 2^b^	7.6	7.1	8.2	7.4	6.9	8.0	7.3	6.8	7.8	7.1	6.7	7.6	<0.001
Red blood cell count (million cells/μL)													
Model 1^a^	4.8	4.7	5.0	4.9	4.7	5.0	4.8	4.7	5.0	4.8	4.6	4.9	0.068
Model 2^c^	4.6	4.5	4.8	4.7	4.5	4.8	4.6	4.5	4.8	4.6	4.5	4.8	0.463
Platelet count (1000 cells/μL)													
Model 1^a^	270.2	253.1	288.2	271.5	254.1	289.9	262.6	246.7	279.4	262.3	244.9	280.6	0.003
Model 2^d^	266.7	250.2	284.0	266.8	250.6	283.8	259.8	245.8	274.3	261.4	246.6	276.9	0.086

Data are back-transformed adjusted means (95% CL). MVPA = moderate-vigorous physical activity. Quartile cut points are 7.0 17.5, 34.3 minutes/day.

^a^ Adjusted for age, sex, race/ethnicity, wear time.

^b^ Adjusted for age, sex, race/ethnicity, wear time, sedentary time, time of year, HEI-2015 score, current illness (flu, pneumonia, or ear infection), asthma, donated blood, poverty income ratio, BMI, smoking status, marital status.

^c^ Adjusted for age, sex, race/ethnicity, wear time, sedentary time, time of year, HEI-2015 score, asthma, anemia, blood transfusion, arthritis, cancer or malignancy, poverty income ratio, BMI, marital status, smoking status.

^d^ Adjusted for age, sex, race/ethnicity, wear time, sedentary time, time of year, HEI-2015 score, current illness (flu, pneumonia, or ear infection), donated blood, blood transfusion, asthma, arthritis, cancer or malignancy, BMI, marital status, smoking status, poverty income ratio.

A significant interaction was observed for WBCs between MVPA and BMI status (p_interaction_<0.001). Higher MVPA was associated with lower WBC counts across all strata of BMI (p_trend_<0.05) with the greatest impact on healthy weight individuals whose average WBC levels were 14% lower in quartile 4 vs. quartile 1 of MVPA. The effects of MVPA on WBC count were less pronounced in overweight (9% lower) and obese (5% lower) individuals when comparing quartile 4 vs. 1 of MVPA. Detailed results for interactions are available in Supplementary Data.

### Associations of total sedentary time with blood cell counts

Results from model 1, adjusting for age, sex, and race/ethnicity, and wear time showed a significant linear association with greater total sedentary time and higher counts of WBCs (p_trend_ = 0.042). There were no significant associations observed for sedentary time with RBCs or platelets. The findings for WBCs were consistent in model 2 (Q4 vs. Q1 mean (95% CI): 7.5 (6.8–8.2) vs. 7.3 (6.7–7.9), which further adjusted for MVPA, time of year, HEI-2015 score, current illness (flu, pneumonia, or ear infection), asthma, donated blood, poverty income ratio, BMI, smoking status, marital status (Table 5).

**Table 5:** Adjusted means (95% CL) for continuous hematologic variables across quartiles of total sedentary time in U.S. adults ≥ 20 years (NHANES 2003–2006).

	Sedentary Time												
	Quartile 1			Quartile 2			Quartile 3			Quartile 4			p_trend_
	Mean	95% CL		Mean	95% CL		Mean	95% CL		Mean	95% CL		
White blood cell count (1000 cells/μL)													
Model 1^a^	6.7	6.3	7.2	6.8	6.3	7.4	6.8	6.3	7.4	6.9	6.3	7.6	0.042
Model 2^b^	7.3	6.7	7.9	7.4	6.9	8.1	7.4	6.8	8.0	7.5	6.8	8.2	0.040
Red blood cell count (million cells/μL)													
Model 1^a^	4.8	4.7	4.9	4.8	4.7	5.0	4.8	4.7	5.0	4.8	4.7	5.0	0.606
Model 2^c^	4.6	4.5	4.8	4.6	4.5	4.8	4.7	4.5	4.8	4.6	4.5	4.8	0.700
Platelet count (1000 cells/μL)													
Model 1^a^	266.2	250.0	283.1	267.1	248.8	286.4	267.0	247.4	287.7	266.2	247.4	286.0	0.999
Model 2^d^	263.3	246.4	281.0	264.3	244.8	285.0	264.3	244.0	285.8	263.4	244.1	283.8	0.984

Data are back-transformed adjusted means (95% CL). MVPA = moderate-vigorous physical activity. Quartile cut points are 389, 472, 553 minutes/day.

^a^ Adjusted for age, sex, race/ethnicity, wear time.

^b^ Adjusted for age, sex, race/ethnicity, wear time, MVPA, time of year, HEI-2015 score, current illness (flu, pneumonia, or ear infection), asthma, donated blood, poverty income ratio, BMI, smoking status, marital status.

^c^ Adjusted for age, sex, race/ethnicity, wear time, MVPA, time of year, HEI-2015 score, asthma, anemia, blood transfusion, arthritis, cancer or malignancy, poverty income ratio, BMI, marital status, smoking status.

^d^ Adjusted for age, sex, race/ethnicity, wear time, MVPA, time of year, HEI-2015 score, current illness (flu, pneumonia, or ear infection), donated blood, blood transfusion, asthma, arthritis, cancer or malignancy, BMI, marital status, smoking status, poverty income ratio.

A significant interaction was observed between sex and sedentary time for WBCs (p_interaction_ = 0.014). Sedentary time was positively associated with WBC count among men (Q4 vs. Q1 mean (95% CI): 7.5 (6.9–8.2) vs. 7.0 (6.5–7.6); p_trend_ = 0.001) but not among women (Q4 vs. Q1 mean (95% CI): 7.4 (6.8–8.0) vs. 7.4 (6.8–8.0); p_trend_ = 0.485). Age group and sedentary time also showed a significant interaction for WBCs (p_interaction_ = 0.005). Higher sedentary time was associated with higher WBC counts across both age groups (p_trend_<0.05). When comparing quartile 4 vs. 1 of sedentary time, individuals ≥50 years of age had 9% higher levels of WBCs compared to 1% in those individuals aged 20–49. Detailed results for interactions are available in Supplementary Data.

## Discussion

Our study shows that blood cell counts, in particular WBCs, differ based on activity level in a nationally representative sample of U.S. adults. This is an important finding as previous research suggests that levels of these cell populations are associated with adverse health outcomes, such as cancer [8, 9]. Levels of WBCs appear to be particularly sensitive to both MVPA and sedentary time in a dose dependent manner. Comprised of five major subtypes (monocytes, lymphocytes, neutrophils, basophils, and eosinophils), WBCs collectively function by mounting an inflammatory response to fight infections and disease. When uncontrolled, this response can initiate an inflammatory state through the release of cytokines and soluble factors that promote the development of disease [25]. After controlling for available covariates known to be associated with altered blood counts (Table 1), our finding that adults with greater MVPA and lower sedentary time have lower counts of WBCs are consistent with other studies that have shown that WBCs are reduced in elderly adult populations who have higher levels of MVPA [26, 27]. Furthermore, our observation that sedentary time had the opposite effect of MVPA, with higher counts of WBCs was similar to a study by Pitsavos *et al*. in which lower levels of self-reported sedentary time was associated with lower WBC levels in adults[28]. Data collected on WBCs collectively suggest that activity may modulate the inflammatory environment by altering the cellular composition in peripheral blood, which may contribute to risk of disease development.

WBCs are not the only cell population in the blood that contribute to inflammation. Platelets play a major role in wound healing through the release of growth factors and inflammatory cytokines that also create a proinflammatory environment conducive for the development of chronic diseases [29]. We observed that greater MVPA was associated with lower platelet counts using model 1, a finding that was similar to research by Wannamethee et al. [30]; however, when controlling for additional covariates in model 2, the relationship with MVPA and platelet levels was no longer significant. To our knowledge, this is the first study to look at these associations in a nationally representative sample across the age spectrum. While our results showed no significant association with platelet counts and sedentary time, sedentary time may affect platelet activation [31], which has been shown to be an important factor in cancer aggressiveness [32]. The biological consequences of alterations to platelet levels require future work that takes into consideration the role of MVPA and sedentary time not only on platelet count but functionality with disease risk.

Conflicting results in the literature have shown MVPA may decrease [33, 34] or increase levels of RBCs [35]. While we observed a borderline significant linear trend in RBCs with greater MVPA using model 1, the trend was not observed after including additional covariates in model 2, suggesting potential confounding by health status or other factors. The previously reported associations between RBCs with MVPA may be the result of acute effects that are not able to be detected in our study design. Other explanations could be due to specific activities the participants were performing or due differences in self-reported data compared to using an accelerometer. As for sedentary time, no associations in RBCs were observed.

Our study possesses many strengths as well as some limitations that must be considered. The strength of our study is that we utilized objectively measured MVPA and sedentary time in a nationally representative population which includes both sexes, a wide age range, and with oversampling of race/ethnicities making our results more generalizable. This is a unique feature as many previous studies have focused on age or disease specific adult populations. While NHANES is cross-sectional in design, limiting our ability to infer any causal relationship, it does have the added benefit of being able to control for a wide range of covariates that may act as confounders in our analysis including dietary factors, smoking, BMI, and illness history. Another limitation is activity levels for individuals are based upon one-week measurement, however this is at the expense of being able to utilize objectively measured levels of MVPA and sedentary time by using accelerometers. Furthermore, accelerometers can underestimate total MVPA as they primarily measure ambulatory motion. However, the benefit of measuring MVPA by accelerometers is added accuracy and elimination of recall bias associated with self-reported data.

## Conclusions

Overall our study suggests that active individuals may maintain a lower inflammatory state through altered levels of WBCs. Thi may contribute to a lower risk of future chronic disease development and provides a potential mechanism to explain the health benefits associated with physical activity. Future work examining if disease risk is mediated through changes in baseline WBCs in individuals with various activity levels is warranted.

## Supporting information

S1 Table Average accelerometer and blood cell counts of the full sample of U.S. adults ≥ 20 years (NHANES 2003–2006). MVPA = moderate-vigorous physical activity. MVPA using 760 accelerometer count threshold. cpm = counts per minute.
(DOCX)

S2 Table Adjusted means (95% CL) for continuous hematologic variables across quartiles of Lifestyle MVPA (760 accelerometer count threshold) in U.S. adults ≥ 20 years (NHANES 2003–2006). Data are back-transformed adjusted means (95% CL). MVPA = moderate-vigorous physical activity. Quartile cut points are 54.3, 83.0, 120.3 minutes/day. ^a^Adjusted for age, sex, race/ethnicity, wear time. ^b^Adjusted for age, sex, race/ethnicity, wear time, time of year, HEI-2015 score, current illness (flu, pneumonia, or ear infection), asthma, donated blood, poverty income ratio, BMI, smoking status, marital status. ^c^Adjusted for age, sex, race/ethnicity, wear time, time of year, HEI-2015 score, asthma, anemia, blood transfusion, arthritis, cancer or malignancy, poverty income ratio, BMI, marital status, smoking status. ^d^Adjusted for age, sex, race/ethnicity, wear time, time of year, HEI-2015 score, current illness (flu, pneumonia, or ear infection), donated blood, blood transfusion, asthma, arthritis, cancer or malignancy, BMI, marital status, smoking status, poverty income ratio.
(DOCX)

S3 Table Adjusted means (95% CL) for continuous hematologic variables across quartiles of counts per minute in U.S. adults ≥ 20 years (NHANES 2003–2006). Data are back-transformed adjusted means (95% CL). Quartile cut points are 216, 304, 412 cpm. ^a^Adjusted for age, sex, race/ethnicity, wear time. ^b^Adjusted for age, sex, race/ethnicity, wear time, time of year, HEI-2015 score, current illness (flu, pneumonia, or ear infection), asthma, donated blood, poverty income ratio, BMI, smoking status, marital status. ^c^Adjusted for age, sex, race/ethnicity, wear time, time of year, HEI-2015 score, asthma, anemia, blood transfusion, arthritis, cancer or malignancy, poverty income ratio, BMI, marital status, smoking status. ^d^Adjusted for age, sex, race/ethnicity, wear time, time of year, HEI-2015 score, current illness (flu, pneumonia, or ear infection), donated blood, blood transfusion, asthma, arthritis, cancer or malignancy, BMI, marital status, smoking status, poverty income ratio.
(DOCX)

S4 Table Adjusted means (95% CL) for continuous hematologic variables across quartiles of MVPA by sex, race/ethnicity, BMI, and age. BMI = Body mass index. ^a^Adjusted for age, sex, race/ethnicity, sedentary time, wear time, time of year, HEI-2015 score, current illness (flu, pneumonia, or ear infection), asthma, donated blood, poverty income ratio, BMI, smoking status, marital status. ^b^Adjusted for age, sex, race/ethnicity, sedentary time, wear time, time of year, HEI-2015 score, asthma, anemia, blood transfusion, arthritis, cancer or malignancy, poverty income ratio, BMI, marital status, smoking status. ^c^Adjusted for age, sex, race/ethnicity, sedentary time, wear time, time of year, HEI-2015 score, current illness (flu, pneumonia, or ear infection), donated blood, blood transfusion, asthma, arthritis, cancer or malignancy, BMI, marital status, smoking status, poverty income ratio.
(DOCX)

S5 Table Adjusted means (95% CL) for continuous hematologic variables across quartiles of total sedentary time by sex, race/ethnicity, BMI, and age. BMI = Body mass index. ^a^Adjusted for age, sex, race/ethnicity, MVPA, wear time, time of year, HEI-2015 score, current illness (flu, pneumonia, or ear infection), asthma, donated blood, poverty income ratio, BMI, smoking status, marital status. ^b^Adjusted for age, sex, race/ethnicity, MVPA, wear time, time of year, HEI-2015 score, asthma, anemia, blood transfusion, arthritis, cancer or malignancy, poverty income ratio, BMI, marital status, smoking status. ^c^Adjusted for age, sex, race/ethnicity, MVPA, wear time, time of year, HEI-2015 score, current illness (flu, pneumonia, or ear infection), donated blood, blood transfusion, asthma, arthritis, cancer or malignancy, BMI, marital status, smoking status, poverty income ratio.
(DOCX)
