# Supplementary material for: Association of physical activity and sedentary time with blood cell counts: National Health and Nutrition Survey 2003-2006
Source: PLoS One. 2018 Sep 25;13(9):e0204277. doi: 10.1371/journal.pone.0204277 (PMC6155506; doi:10.1371/journal.pone.0204277)
Supplement: S1 Table — MVPA = moderate-vigorous physical activity. MVPA using 760 accelerometer count threshold. cpm = counts per minute. (DOCX) [file pone.0204277.s001.docx]

**S1 Table.** Average accelerometer and blood cell counts of the full sample of U.S. adults ≥ 20 years (NHANES 2003-2006)

|  | **Full Sample (n = 4857)** | | |  |
| --- | --- | --- | --- | --- |
|  | **Mean** | **95% CL** | | |
| **Accelerometer variables** |  |  |  | |
| Wear time (minutes/day) | 880.5 | 875.6 | 885.4 | |
| Counts per minute (cpm) | 341.8 | 334.3 | 349.3 | |
| **MVPA** |  |  |  | |
| *Quartile 1 (≤ 54.3 minutes/day)* | 36.5 | 35.5 | 37.4 | |
| *Quartile 2 (54.4 to 83.0 minutes /day)* | 68.8 | 68.3 | 69.3 | |
| *Quartile 3 (83.1 to 120.3 minutes /day)* | 99.7 | 99.0 | 100.3 | |
| *Quartile 4 (≥120.4 minutes /day)* | 158.7 | 156.3 | 161.0 | |
| **Sedentary Time** |  |  |  | |
| *Quartile 1 (≤ 216 cpm)* | 160.9 | 157.8 | 164.0 | |
| *Quartile 2 (217 to 304 cpm)* | 261.9 | 260.1 | 263.7 | |
| *Quartile 3 (305 to 412 cpm)* | 355.0 | 352.8 | 357.3 | |
| *Quartile 4 (≥413 cpm)* | 545.0 | 537.3 | 552.8 | |
|  |  |  |  | |
| **Blood Cell Types** |  |  |  | |
| White blood cell count (1000 cells/µL) | 7.3 | 7.2 | 7.4 | |
| Red blood cell count (million cells/µL) | 4.8 | 4.8 | 4.8 | |
| Platelet count (1000 cells/µL) | 273.4 | 270.9 | 276.0 | |

MVPA = moderate-vigorous physical activity. MVPA using 760 accelerometer count threshold. cpm = counts per minute.
