# Supplementary material for: Association of physical activity and sedentary time with blood cell counts: National Health and Nutrition Survey 2003-2006
Source: PLoS One. 2018 Sep 25;13(9):e0204277. doi: 10.1371/journal.pone.0204277 (PMC6155506; doi:10.1371/journal.pone.0204277)
Supplement: S3 Table — Data are back-transformed adjusted means (95% CL). Quartile cut points are 216, 304, 412 cpm. aAdjusted for age, sex, race/ethnicity, wear time. bAdjusted for age, sex, race/ethnicity, wear time, time of year, HEI-2015 score, current illness (flu, pneumonia, or ear infection), asthma, donated blood, poverty income ratio, BMI, smoking status, marital status. cAdjusted for age, sex, race/ethnicity, wear time, time of year, HEI-2015 score, asthma, anemia, blood transfusion, arthritis, cancer or malignancy, poverty income ratio, BMI, marital status, smoking status. dAdjusted for age, sex, race/ethnicity, wear time, time of year, HEI-2015 score, current illness (flu, pneumonia, or ear infection), donated blood, blood transfusion, asthma, arthritis, cancer or malignancy, BMI, marital status, smoking status, poverty income ratio. (DOCX) [file pone.0204277.s003.docx]

**S3 Table.** Adjusted means (95% CL) for continuous hematologic variables across quartiles of counts per minute in U.S. adults ≥ 20 years (NHANES 2003-2006)

|  | **Counts per Minute** | | | | | | | | | | | | | | |  |
| --- | --- | --- | --- | --- | --- | --- | --- | --- | --- | --- | --- | --- | --- | --- | --- | --- |
|  | **Quartile 1** | | |  | **Quartile 2** | | |  | **Quartile 3** | | |  | **Quartile 4** | | | **p_trend_** |
|  | **Mean** | **95% CL** | |  | **Mean** | **95% CL** | |  | **Mean** | **95% CL** | |  | **Mean** | **95% CL** | |  |
| White blood cell count (1000 cells/µL) |  |  |  |  |  |  |  |  |  |  |  |  |  |  |  |  |
| Model 1^a^ | 7.1 | 6.8 | 7.5 |  | 6.9 | 6.7 | 7.2 |  | 6.7 | 6.5 | 7.0 |  | 6.5 | 6.3 | 6.7 | <0.001 |
| Model 2^b^ | 7.6 | 7.2 | 8.0 |  | 7.5 | 7.2 | 7.8 |  | 7.3 | 6.9 | 7.7 |  | 7.2 | 6.9 | 7.5 | 0.001 |
| Red blood cell count (million cells/µL) |  |  |  |  |  |  |  |  |  |  |  |  |  |  |  |  |
| Model 1^a^ | 4.8 | 4.8 | 4.9 |  | 4.9 | 4.8 | 4.9 |  | 4.8 | 4.8 | 4.9 |  | 4.8 | 4.7 | 4.8 | 0.078 |
| Model 2^c^ | 4.6 | 4.6 | 4.7 |  | 4.7 | 4.6 | 4.7 |  | 4.7 | 4.6 | 4.7 |  | 4.6 | 4.5 | 4.7 | 0.310 |
| Platelet count (1000 cells/µL) |  |  |  |  |  |  |  |  |  |  |  |  |  |  |  |  |
| Model 1^a^ | 268.2 | 264.5 | 271.9 |  | 269.8 | 266.2 | 273.4 |  | 263.0 | 259.9 | 266.1 |  | 265.4 | 260.6 | 270.3 | 0.131 |
| Model 2^d^ | 263.9 | 254.7 | 273.2 |  | 266.3 | 257.3 | 275.7 |  | 260.5 | 250.3 | 270.9 |  | 265.4 | 255.8 | 275.2 | 0.877 |

Data are back-transformed adjusted means (95% CL). Quartile cut points are 216, 304, 412 cpm.

^a^ Adjusted for age, sex, race/ethnicity, wear time.

^b^ Adjusted for age, sex, race/ethnicity, wear time, time of year, HEI-2015 score, current illness (flu, pneumonia, or ear infection), asthma, donated blood, poverty income ratio, BMI, smoking status, marital status.

^c^ Adjusted for age, sex, race/ethnicity, wear time, time of year, HEI-2015 score, asthma, anemia, blood transfusion, arthritis, cancer or malignancy, poverty income ratio, BMI, marital status, smoking status.

^d^ Adjusted for age, sex, race/ethnicity, wear time, time of year, HEI-2015 score, current illness (flu, pneumonia, or ear infection), donated blood, blood transfusion, asthma, arthritis, cancer or malignancy, BMI, marital status, smoking status, poverty income ratio.
