# Supplementary material for: Association of physical activity and sedentary time with blood cell counts: National Health and Nutrition Survey 2003-2006
Source: PLoS One. 2018 Sep 25;13(9):e0204277. doi: 10.1371/journal.pone.0204277 (PMC6155506; doi:10.1371/journal.pone.0204277)
Supplement: S4 Table — BMI = Body mass index. aAdjusted for age, sex, race/ethnicity, sedentary time, wear time, time of year, HEI-2015 score, current illness (flu, pneumonia, or ear infection), asthma, donated blood, poverty income ratio, BMI, smoking status, marital status. bAdjusted for age, sex, race/ethnicity, sedentary time, wear time, time of year, HEI-2015 score, asthma, anemia, blood transfusion, arthritis, cancer or malignancy, poverty income ratio, BMI, marital status, smoking status. cAdjusted for age, sex, race/ethnicity, sedentary time, wear time, time of year, HEI-2015 score, current illness (flu, pneumonia, or ear infection), donated blood, blood transfusion, asthma, arthritis, cancer or malignancy, BMI, marital status, smoking status, poverty income ratio. (DOCX) [file pone.0204277.s004.docx]

**S4 Table.**  Adjusted means (95% CL) for continuous hematologic variables across quartiles of MVPA by sex, race/ethnicity, BMI, and age.

|  | **MVPA** | | | | | | | | | | | | | |
| --- | --- | --- | --- | --- | --- | --- | --- | --- | --- | --- | --- | --- | --- | --- |
|  | **White blood cell count (1000 cells/µL)** | | | | | | | | | | | |  |  |
|  | **Quartile 1** | | | **Quartile 2** | | | **Quartile 3** | | | **Quartile 4** | | |  |  |
|  | **mean** | **95% CI** | | **mean** | **95% CI** | | **mean** | **95% CI** | | **mean** | **95% CI** | | **p_trend_** | **p_interaction_** |
| **White blood cell count (1000 cells/µL)^a^** |  |  |  |  |  |  |  |  |  |  |  |  |  |  |
| Sex |  |  |  |  |  |  |  |  |  |  |  |  |  | 0.358 |
| *Males (n=3030)* | 7.7 | 7.1 | 8.4 | 7.3 | 6.8 | 7.8 | 7.2 | 6.7 | 7.7 | 7.1 | 6.6 | 7.6 | 0.003 |  |
| *Females (n=1827)* | 7.5 | 7.0 | 8.1 | 7.5 | 6.9 | 8.0 | 7.4 | 6.8 | 7.9 | 7.1 | 6.7 | 7.7 | 0.000 |  |
| Race/Ethnicity |  |  |  |  |  |  |  |  |  |  |  |  |  | 0.649 |
| *Non-Hispanic White (n=2520)* | 7.9 | 7.3 | 8.5 | 7.6 | 7.1 | 8.1 | 7.5 | 7.1 | 8.0 | 7.3 | 6.9 | 7.8 | 0.002 |  |
| *Non-Hispanic Black (n=1020)* | 6.6 | 6.2 | 7.1 | 6.4 | 5.9 | 6.9 | 6.2 | 5.8 | 6.7 | 6.3 | 5.9 | 6.6 | 0.008 |  |
| *Hispanic (n=973)* | 8.0 | 7.4 | 8.8 | 8.0 | 7.4 | 8.6 | 7.9 | 7.2 | 8.5 | 7.6 | 7.0 | 8.2 | 0.296 |  |
| BMI |  |  |  |  |  |  |  |  |  |  |  |  |  | <0.001 |
| *Healthy weight (18.5-24.9 kg/m^2^; n=1728)* | 7.6 | 7.1 | 8.2 | 6.9 | 6.5 | 7.3 | 6.8 | 6.4 | 7.2 | 6.5 | 6.1 | 7.0 | <0.001 |  |
| *Overweight (25.0-29.9 kg/m^2^; n=1417)* | 7.5 | 7.0 | 7.9 | 7.2 | 6.8 | 7.6 | 7.1 | 6.7 | 7.1 | 6.8 | 7.5 | 6.8 | 0.033 |  |
| *Obese (≥30.0 kg/m^2^; n=1643)* | 7.9 | 7.3 | 8.4 | 7.9 | 7.4 | 8.5 | 7.7 | 7.3 | 8.2 | 7.5 | 7.1 | 7.9 | 0.003 |  |
| *Age Group* |  |  |  |  |  |  |  |  |  |  |  |  |  | 0.578 |
| *20-49 years (n=2918)* | 7.7 | 7.2 | 8.3 | 7.5 | 7.1 | 8.0 | 7.4 | 7.0 | 7.9 | 7.3 | 6.9 | 7.8 | 0.009 |  |
| *≥50 years (n=1939)* | 7.4 | 6.8 | 8.0 | 7.1 | 6.7 | 7.6 | 7.0 | 6.5 | 7.5 | 6.8 | 6.3 | 7.3 | 0.001 |  |
|  |  |  |  |  |  |  |  |  |  |  |  |  |  |  |
| **Red blood cell count (million cells/µL)^b^** |  |  |  |  |  |  |  |  |  |  |  |  |  |  |
| Sex |  |  |  |  |  |  |  |  |  |  |  |  |  | <0.001 |
| *Males (n=3030)* | 4.8 | 4.7 | 4.9 | 4.8 | 4.7 | 5.0 | 4.9 | 4.7 | 5.0 | 4.8 | 4.7 | 5.0 | 0.741 |  |
| *Females (n=1827)* | 4.3 | 4.2 | 4.4 | 4.3 | 4.2 | 4.5 | 4.3 | 4.1 | 4.4 | 4.2 | 4.1 | 4.4 | 0.271 |  |
| Race/Ethnicity |  |  |  |  |  |  |  |  |  |  |  |  |  | 0.045 |
| *Non-Hispanic White (n=2520)* | 4.6 | 4.5 | 4.7 | 4.6 | 4.5 | 4.8 | 4.6 | 4.5 | 4.8 | 4.6 | 4.4 | 4.7 | 0.272 |  |
| *Non-Hispanic Black (n=1020)* | 4.5 | 4.4 | 4.6 | 4.6 | 4.4 | 4.7 | 4.6 | 4.5 | 4.8 | 4.6 | 4.5 | 4.8 | 0.145 |  |
| *Hispanic (n=973)* | 4.7 | 4.5 | 4.8 | 4.7 | 4.5 | 4.8 | 4.6 | 4.5 | 4.8 | 4.6 | 4.5 | 4.8 | 0.569 |  |
| BMI |  |  |  |  |  |  |  |  |  |  |  |  |  | 0.386 |
| *Healthy weight (18.5-24.9 kg/m^2^; n=1728)* | 4.5 | 4.4 | 4.6 | 4.5 | 4.4 | 4.7 | 4.5 | 4.4 | 4.7 | 4.5 | 4.3 | 4.6 | 0.464 |  |
| *Overweight (25.0-29.9 kg/m^2^; n=1417)* | 4.6 | 4.5 | 4.7 | 4.6 | 4.5 | 4.8 | 4.6 | 4.5 | 4.8 | 4.6 | 4.5 | 4.8 | 0.874 |  |
| *Obese (≥30.0 kg/m^2^; n=1643)* | 4.7 | 4.5 | 4.8 | 4.7 | 4.5 | 4.8 | 4.7 | 4.5 | 4.8 | 4.7 | 4.5 | 4.8 | 0.605 |  |
| Age Group |  |  |  |  |  |  |  |  |  |  |  |  |  | 0.063 |
| *20-49 years (n=2918)* | 4.6 | 4.5 | 4.8 | 4.7 | 4.5 | 4.8 | 4.6 | 4.5 | 4.8 | 4.6 | 4.5 | 4.8 | 0.376 |  |
| *≥50 years (n=1939)* | 4.5 | 4.3 | 4.6 | 4.6 | 4.4 | 4.7 | 4.6 | 4.4 | 4.7 | 4.6 | 4.4 | 4.7 | 0.010 |  |
|  |  |  |  |  |  |  |  |  |  |  |  |  |  |  |
| **Platelet count (1000 cells/µL)^c^** |  |  |  |  |  |  |  |  |  |  |  |  |  |  |
| Sex |  |  |  |  |  |  |  |  |  |  |  |  |  | 0.046 |
| *Males (n=3030)* | 248 | 230 | 269 | 258 | 239 | 278 | 252 | 232 | 272 | 253 | 234 | 273 | 0.949 |  |
| *Females (n=1827)* | 297 | 275 | 320 | 288 | 267 | 310 | 283 | 263 | 304 | 283 | 262 | 304 | 0.134 |  |
| Race/Ethnicity |  |  |  |  |  |  |  |  |  |  |  |  |  | 0.619 |
| *Non-Hispanic White (n=2520)* | 265 | 247 | 284 | 264 | 245 | 284 | 257 | 239 | 277 | 259 | 241 | 279 | 0.258 |  |
| *Non-Hispanic Black (n=1020)* | 259 | 239 | 281 | 263 | 242 | 285 | 255 | 234 | 276 | 255 | 235 | 277 | 0.796 |  |
| *Hispanic (n=973)* | 257 | 233 | 282 | 264 | 243 | 287 | 263 | 241 | 285 | 261 | 241 | 282 | 0.765 |  |
| BMI |  |  |  |  |  |  |  |  |  |  |  |  |  | 0.010 |
| *Healthy weight (18.5-24.9 kg/m^2^; n=1728)* | 271 | 252 | 292 | 258 | 239 | 279 | 253 | 234 | 274 | 258 | 238 | 279 | 0.109 |  |
| *Overweight (25.0-29.9 kg/m^2^; n=1417)* | 263 | 243 | 284 | 269 | 249 | 292 | 260 | 241 | 280 | 264 | 245 | 283 | 0.167 |  |
| *Obese (≥30.0 kg/m^2^; n=1643)* | 271 | 251 | 293 | 278 | 258 | 299 | 273 | 253 | 294 | 267 | 247 | 288 | 0.819 |  |
| Age Group |  |  |  |  |  |  |  |  |  |  |  |  |  | 0.056 |
| *20-49 years (n=2918)* | 278 | 256 | 300 | 272 | 252 | 292 | 266 | 248 | 286 | 268 | 248 | 288 | 0.029 |  |
| *≥50 years (n=1939)* | 250 | 233 | 268 | 259 | 241 | 278 | 255 | 236 | 274 | 258 | 241 | 276 | 0.235 |  |

BMI = Body mass index

^a^ Adjusted for age, sex, race/ethnicity, sedentary time, wear time, time of year, HEI-2015 score, current illness (flu, pneumonia, or ear infection), asthma, donated blood, poverty income ratio, BMI, smoking status, marital status.

^b^ Adjusted for age, sex, race/ethnicity, sedentary time, wear time, time of year, HEI-2015 score, asthma, anemia, blood transfusion, arthritis, cancer or malignancy, poverty income ratio, BMI, marital status, smoking status.

^c^ Adjusted for age, sex, race/ethnicity, sedentary time, wear time, time of year, HEI-2015 score, current illness (flu, pneumonia, or ear infection), donated blood, blood transfusion, asthma, arthritis, cancer or malignancy, BMI, marital status, smoking status, poverty income ratio.
