# Supplementary material for: Association of physical activity and sedentary time with blood cell counts: National Health and Nutrition Survey 2003-2006
Source: PLoS One. 2018 Sep 25;13(9):e0204277. doi: 10.1371/journal.pone.0204277 (PMC6155506; doi:10.1371/journal.pone.0204277)
Supplement: S5 Table — BMI = Body mass index. aAdjusted for age, sex, race/ethnicity, MVPA, wear time, time of year, HEI-2015 score, current illness (flu, pneumonia, or ear infection), asthma, donated blood, poverty income ratio, BMI, smoking status, marital status. bAdjusted for age, sex, race/ethnicity, MVPA, wear time, time of year, HEI-2015 score, asthma, anemia, blood transfusion, arthritis, cancer or malignancy, poverty income ratio, BMI, marital status, smoking status. cAdjusted for age, sex, race/ethnicity, MVPA, wear time, time of year, HEI-2015 score, current illness (flu, pneumonia, or ear infection), donated blood, blood transfusion, asthma, arthritis, cancer or malignancy, BMI, marital status, smoking status, poverty income ratio. (DOCX) [file pone.0204277.s005.docx]

**S5 Table.**  Adjusted means (95% CL) for continuous hematologic variables across quartiles of total sedentary time by sex, race/ethnicity, BMI, and age.

|  | **Sedentary Time** | | | | | | | | | | | | | |
| --- | --- | --- | --- | --- | --- | --- | --- | --- | --- | --- | --- | --- | --- | --- |
|  | **White blood cell count (1000 cells/µL)** | | | | | | | | | | | |  |  |
|  | **Quartile 1** | | | **Quartile 2** | | | **Quartile 3** | | | **Quartile 4** | | |  |  |
|  | **mean** | **95% CI** | | **mean** | **95% CI** | | **mean** | **95% CI** | | **mean** | **95% CI** | | **p_trend_** | **p_interaction_** |
| **White blood cell count (1000 cells/µL)^a^** |  |  |  |  |  |  |  |  |  |  |  |  |  |  |
| Sex |  |  |  |  |  |  |  |  |  |  |  |  |  | 0.014 |
| *Males (n=3030)* | 7.0 | 6.5 | 7.6 | 7.2 | 6.8 | 7.7 | 7.4 | 6.9 | 7.9 | 7.5 | 6.9 | 8.2 | 0.001 |  |
| *Females (n=1827)* | 7.4 | 6.8 | 8.0 | 7.5 | 6.9 | 8.2 | 7.3 | 6.8 | 7.9 | 7.4 | 6.8 | 8.0 | 0.485 |  |
| Race/Ethnicity |  |  |  |  |  |  |  |  |  |  |  |  |  | 0.879 |
| *Non-Hispanic White (n=2520)* | 7.4 | 6.9 | 8.0 | 7.6 | 7.1 | 8.2 | 7.6 | 7.1 | 8.2 | 7.8 | 7.2 | 8.4 | 0.104 |  |
| *Non-Hispanic Black (n=1020)* | 6.3 | 5.9 | 6.8 | 6.4 | 6.0 | 6.9 | 6.3 | 5.9 | 6.8 | 6.5 | 6.1 | 7.0 | 0.115 |  |
| *Hispanic (n=973)* | 7.7 | 7.1 | 8.5 | 7.8 | 7.3 | 8.4 | 7.9 | 7.3 | 8.5 | 8.2 | 7.4 | 9.0 | 0.020 |  |
| BMI |  |  |  |  |  |  |  |  |  |  |  |  |  | 0.225 |
| *Healthy weight (18.5-24.9 kg/m^2^; n=1728)* | 7.3 | 6.9 | 7.6 | 7.2 | 6.8 | 7.8 | 7.3 | 6.9 | 7.8 | 7.7 | 7.3 | 8.1 | 0.054 |  |
| *Overweight (25.0-29.9 kg/m^2^; n=1417)* | 7.2 | 6.9 | 7.4 | 7.3 | 7.0 | 7.7 | 7.2 | 6.8 | 7.6 | 7.4 | 7.1 | 7.8 | 0.247 |  |
| *Obese (≥30.0 kg/m^2^; n=1643)* | 6.9 | 6.5 | 7.4 | 7.2 | 6.7 | 7.7 | 7.1 | 6.7 | 7.5 | 7.2 | 6.8 | 7.6 | 0.289 |  |
| Age Group |  |  |  |  |  |  |  |  |  |  |  |  |  | 0.005 |
| *20-49 years (n=2918)* | 7.4 | 7.9 | 6.9 | 7.4 | 8.0 | 6.9 | 7.3 | 7.7 | 6.9 | 7.5 | 8.1 | 6.9 | 0.031 |  |
| *≥50 years (n=1939)* | 6.7 | 7.2 | 6.2 | 7.1 | 7.6 | 6.6 | 7.1 | 7.7 | 6.6 | 7.3 | 7.9 | 6.7 | 0.009 |  |
|  |  |  |  |  |  |  |  |  |  |  |  |  |  |  |
| **Red blood cell count (million cells/µL)^b^** |  |  |  |  |  |  |  |  |  |  |  |  |  |  |
| Sex |  |  |  |  |  |  |  |  |  |  |  |  |  | 0.390 |
| *Males (n=3030)* | 4.8 | 4.7 | 5.0 | 4.8 | 4.7 | 5.0 | 4.8 | 4.7 | 4.9 | 4.8 | 4.6 | 4.9 | 0.554 |  |
| *Females (n=1827)* | 4.3 | 4.1 | 4.4 | 4.3 | 4.2 | 4.4 | 4.3 | 4.2 | 4.4 | 4.3 | 4.1 | 4.4 | 0.980 |  |
| Race/Ethnicity |  |  |  |  |  |  |  |  |  |  |  |  |  | 0.133 |
| *Non-Hispanic White (n=2520)* | 4.6 | 4.4 | 4.7 | 4.6 | 4.5 | 4.8 | 4.6 | 4.5 | 4.8 | 4.6 | 4.4 | 4.7 | 0.760 |  |
| *Non-Hispanic Black (n=1020)* | 4.6 | 4.4 | 4.7 | 4.6 | 4.5 | 4.8 | 4.6 | 4.4 | 4.7 | 4.5 | 4.3 | 4.7 | 0.049 |  |
| *Hispanic (n=973)* | 4.7 | 4.5 | 4.8 | 4.7 | 4.5 | 4.8 | 4.6 | 4.5 | 4.8 | 4.7 | 4.5 | 4.9 | 0.744 |  |
| BMI |  |  |  |  |  |  |  |  |  |  |  |  |  | 0.274 |
| *Healthy weight (18.5-24.9 kg/m^2^; n=1728)* | 4.5 | 4.3 | 4.6 | 4.5 | 4.4 | 4.6 | 4.4 | 4.3 | 4.5 | 4.4 | 4.3 | 4.5 | 0.103 |  |
| *Overweight (25.0-29.9 kg/m^2^; n=1417)* | 4.6 | 4.5 | 4.7 | 4.6 | 4.4 | 4.7 | 4.6 | 4.5 | 4.7 | 4.6 | 4.4 | 4.7 | 0.526 |  |
| *Obese (≥30.0 kg/m^2^; n=1643)* | 4.7 | 4.6 | 4.8 | 4.7 | 4.6 | 4.8 | 4.7 | 4.6 | 4.8 | 4.7 | 4.6 | 4.8 | 0.753 |  |
| Age Group |  |  |  |  |  |  |  |  |  |  |  |  |  | 0.001 |
| *20-49 years (n=2918)* | 4.6 | 4.5 | 4.7 | 4.6 | 4.5 | 4.7 | 4.6 | 4.5 | 4.7 | 4.6 | 4.5 | 4.7 | 0.358 |  |
| *≥50 years (n=1939)* | 4.5 | 4.4 | 4.7 | 4.6 | 4.4 | 4.7 | 4.5 | 4.3 | 4.6 | 4.4 | 4.3 | 4.6 | 0.014 |  |
|  |  |  |  |  |  |  |  |  |  |  |  |  |  |  |
| **Platelet count (1000 cells/µL)^c^** |  |  |  |  |  |  |  |  |  |  |  |  |  |  |
| Sex |  |  |  |  |  |  |  |  |  |  |  |  |  | 0.057 |
| *Males (n=3030)* | 291 | 267 | 316 | 285 | 262 | 311 | 283 | 257 | 311 | 289 | 267 | 312 | 0.181 |  |
| *Females (n=1827)* | 249 | 227 | 272 | 253 | 231 | 276 | 255 | 233 | 278 | 252 | 231 | 273 | 0.571 |  |
| Race/Ethnicity |  |  |  |  |  |  |  |  |  |  |  |  |  | 0.671 |
| *Non-Hispanic White (n=2520)* | 259 | 238 | 281 | 261 | 239 | 284 | 262 | 240 | 285 | 263 | 241 | 287 | 0.657 |  |
| *Non-Hispanic Black (n=1020)* | 261 | 238 | 285 | 256 | 234 | 281 | 259 | 237 | 284 | 256 | 230 | 284 | 0.849 |  |
| *Hispanic (n=973)* | 263 | 241 | 286 | 261 | 238 | 285 | 261 | 236 | 288 | 261 | 235 | 291 | 0.657 |  |
| BMI |  |  |  |  |  |  |  |  |  |  |  |  |  | 0.605 |
| *Healthy weight (18.5-24.9 kg/m^2^; n=1728)* | 256 | 242 | 272 | 253 | 237 | 269 | 251 | 236 | 266 | 256 | 238 | 274 | 0.860 |  |
| *Overweight (25.0-29.9 kg/m^2^; n=1417)* | 269 | 256 | 282 | 269 | 255 | 284 | 268 | 257 | 281 | 269 | 255 | 283 | 0.974 |  |
| *Obese (≥30.0 kg/m^2^; n=1643)* | 262 | 249 | 275 | 263 | 249 | 278 | 268 | 254 | 283 | 271 | 258 | 284 | 0.042 |  |
| Age Group |  |  |  |  |  |  |  |  |  |  |  |  |  | 0.002 |
| *20-49 years (n=2918)* | 267 | 246 | 289 | 269 | 246 | 293 | 269 | 246 | 293 | 265 | 241 | 291 | 0.943 |  |
| *≥50 years (n=1939)* | 258 | 238 | 280 | 249 | 229 | 271 | 250 | 228 | 273 | 255 | 233 | 278 | 0.904 |  |

BMI = Body mass index

^a^ Adjusted for age, sex, race/ethnicity, MVPA, wear time, time of year, HEI-2015 score, current illness (flu, pneumonia, or ear infection), asthma, donated blood, poverty income ratio, BMI, smoking status, marital status.

^b^ Adjusted for age, sex, race/ethnicity, MVPA, wear time, time of year, HEI-2015 score, asthma, anemia, blood transfusion, arthritis, cancer or malignancy, poverty income ratio, BMI, marital status, smoking status.

^c^ Adjusted for age, sex, race/ethnicity, MVPA, wear time, time of year, HEI-2015 score, current illness (flu, pneumonia, or ear infection), donated blood, blood transfusion, asthma, arthritis, cancer or malignancy, BMI, marital status, smoking status, poverty income ratio.
